# The WOMAN Trial (World Maternal Antifibrinolytic Trial): tranexamic acid for the treatment of postpartum haemorrhage: an international randomised, double blind placebo controlled trial

**DOI:** 10.1186/1745-6215-11-40

**Published:** 2010-04-16

**Authors:** Haleema Shakur, Diana Elbourne, Metin Gülmezoglu, Zarko Alfirevic, Carine Ronsmans, Elizabeth Allen, Ian Roberts

**Affiliations:** 1 Clinical Trials Unit, London School of Hygiene & Tropical Medicine, Keppel Street, London WC1E 7HT, UK; 2 Department of Reproductive Health and Research, World Health Organization, Avenue Appia 20, CH-1211 Geneva 27, Switzerland; 3 Division of Perinatal and Reproductive Medicine, University of Liverpool, Liverpool Women's Hospital, Crown Street, Liverpool L8 7SS, UK; 4 Medical Statistics Unit, London School of Hygiene & Tropical Medicine, Keppel Street, London WC1E 7HT, UK; 5 Infectious Diseases Epidemiology Unit, London School of Hygiene & Tropical Medicine, Keppel Street, London WC1E 7HT, UK

## Abstract

**Background:**

Each year, worldwide about 530,000 women die from causes related to pregnancy and childbirth. Of the deaths 99% are in low and middle income countries. Obstetric haemorrhage is the leading cause of maternal mortality, most occurring in the postpartum period. Systemic antifibrinolytic agents are widely used in surgery to prevent clot breakdown (fibrinolysis) in order to reduce surgical blood loss. At present there is little reliable evidence from randomised trials on the effectiveness of tranexamic acid in the treatment of postpartum haemorrhage.

**Methods:**

The Trial aims to determine the effect of early administration of tranexamic acid on mortality, hysterectomy and other morbidities (surgical interventions, blood transfusion, risk of non-fatal vascular events) in women with clinically diagnosed postpartum haemorrhage. The use of health services and safety, especially thromboembolic effect, on breastfed babies will also be assessed. The trial will be a large, pragmatic, randomised, double blind, placebo controlled trial among 15,000 women with a clinical diagnosis of postpartum haemorrhage. All legally adult women with clinically diagnosed postpartum haemorrhage following vaginal delivery of a baby or caesarean section will potentially be eligible. The fundamental eligibility criterion is the responsible clinician's 'uncertainty' as to whether or not to use an antifibrinolytic agent in a particular woman with postpartum haemorrhage. Treatment will entail a dose of tranexamic acid (1 gram by intravenous injection) or placebo (sodium chloride 0.9%) will be given as soon as possible after randomisation. A second dose may be given if after 30 minutes bleeding continues, or if it stops and restarts within 24 hours after the first dose.

The main analyses will be on an 'intention to treat' basis, irrespective of whether the allocated treatment was received or not. Subgroup analyses for the primary outcome will be based on type of delivery; administration or not of prophylactic uterotonics; and on whether the clinical decision to consider trial entry was based primarily on estimated blood loss alone or on haemodynamic instability. A study with 15,000 women will have over 90% power to detect a 25% reduction from 4% to 3% in the primary endpoint of mortality or hysterectomy.

**Trial registration:**

Current Controlled Trials: ISRCTN76912190 and Clinicaltrials.gov ID: NCT00872469

## Background

Each year, worldwide, about 530,000 women die from causes related to pregnancy and childbirth. Nearly all (99%) of these deaths are in low and middle income countries[1]. Haemorrhage, which usually occurs in the postpartum period, is responsible for between one quarter and one third of obstetric deaths[2]. Postpartum haemorrhage (PPH) is commonly defined as blood loss of ≥ 500 mL after vaginal delivery of a baby, or ≥ 1000 mL after caesarean section. However, these thresholds do not take into account pre-existing health status, and blood loss of as little as 200 mL can be life-threatening for a woman with severe anaemia or cardiac disease[3].

Of the 14 million women who have PPH each year, about 2% die, with an average interval from onset of bleeding to death of 2 to 4 hours[2]. Although many deaths from PPH occur outside healthcare facilities, a significant number occur in hospital, where effective emergency care has the potential to save lives [4,5]. PPH is also an important cause of maternal mortality in high income countries, accounting for about 13% of maternal deaths[6].

PPH also causes hospital morbidity. Many women require blood transfusion which sometimes can transmit blood borne viral infections. Approximately 1% of women with spontaneous vaginal deliveries require transfusion, but the figure increases to 5% or 6% for women with instrumental deliveries or caesarean sections [7]. The risk of infection from transfused blood is considerably higher in countries that do not screen all blood for transfusion[8]. In high income countries the risk of transfusion transmitted infection is low, but adverse reactions related to blood transfusion are common[9].

Severe anaemia is a common consequence of PPH and affects about 11% of the 14 million women with PPH each year[10]. Severe anaemia can cause disabling fatigue and seriously reduce a woman's capacity to look after her children and to work[11]. Systemic antifibrinolytic agents are widely used in surgery to prevent clot breakdown (fibrinolysis) in order to reduce surgical blood loss. A systematic review of randomised controlled trials of antifibrinolytic agents in surgical patients identified 211 randomised controlled trials including 20,781 randomised participants. The results show that tranexamic acid (TXA) reduces the risk of blood transfusion by a relative 39% (RR = 0.61, 95% CI 0.54 to 0.69). In all patients, TXA reduces transfused volume by 1.1 units (95% CI 0.64 to 1.59). TXA may also reduce the need for re-operation due to bleeding (RR = 0.67, 95% CI 0.41 to 1.09). There was no evidence of an increased risk of thrombotic events[12].

TXA significantly reduces uterine blood loss in women with menorrhagia and is "recommended for consideration" as a treatment in intractable postpartum haemorrhage in the UK[13]. However, at present there is little reliable evidence from randomised trials on the effectiveness of TXA in the treatment of PPH. A systematic review of randomised trials of TXA in PPH conducted by the investigators identified three trials of the *prophylactic *use of TXA, including a total of 460 participants[14]. Although there was a statistically significant reduction in average postpartum blood loss in women treated with TXA [weighted mean reduction of approximately 100 mL] the quality of the trials was poor. None had adequate allocation concealment and even in aggregate the trials were too small to assess the effects of TXA on the clinically important end points of mortality, hysterectomy and thrombotic side effects. The most recently updated PPH treatment guidelines prepared by the World Health Organization (WHO) state that TXA may be used in the treatment of PPH if other measures fail, but points out that the quality of evidence on which this recommendation is based is low and recommends that further clinical trials of TXA in PPH are conducted.

### Need for a trial

The WOMAN Trial will provide a reliable scientific basis for recommendations as to whether or not tranexamic acid should be used in the treatment of PPH. If TXA reduces mortality in women with PPH, this would be of considerable significance worldwide. There is a global commitment to the Millennium Development Goal (MDG) of reducing maternal deaths by three-quarters by the year 2015, a commitment that requires a reduction of the maternal mortality ratio by 5.5% each year. Because maternal haemorrhage accounts for over a quarter of deaths, an effective treatment for PPH would contribute importantly to the MDG of reducing maternal mortality. TXA might also reduce the need for hysterectomy, decrease the risk of anaemia and avoid the need for blood transfusion. Blood is a scarce resource in many countries with a risk of transfusion transmitted infections. If TXA was effective in the hospital setting, further research could be conducted to evaluate its use in the community, possibly including the use of oral rather than intravenous administration.

The results of this trial will be disseminated by publication in peer reviewed medical journals, conference presentations, and in an updated version of the Cochrane systematic review of treatments for postpartum bleeding. There is evidence that hospitals participating in multi-centre trials are more likely to implement the trial results[15]. For this reason, a large international multi-centre trial like the WOMAN trial can be expected to have a substantial impact on clinical practice. The large network of collaborating sites will ensure that the results are disseminated worldwide.

### Tranexamic acid and its effect on bleeding

In the haemostatic process, coagulation occurs rapidly at the site of a damaged vessel building a tight net of fibrin, while at the same time, the fibrinolytic system removes the fibrin deposits that could cause permanent vascular occlusion once vascular repair has taken place[16]. The coagulation and fibrinolytic system are believed to be in a state of dynamic balance which maintains an intact vascular system. Tranexamic acid is a potent antifibrinolytic agent that exerts its effect by blocking lysine binding sites on plasminogen molecules and has the potential to enhance the effectiveness of the patient's own haemostatic mechanisms. Consequently, clot breakdown (fibrinolysis) is inhibited and excessive or recurrent bleeding is reduced.

During delivery, when the placenta separates from the uterine wall, a sequence of physiologic and haemostatic changes occur that reduce bleeding: strong myometrial contractions, increased platelet activity, a massive release of coagulant factors and a parallel increase in the fibrinolytic activity[17]. As a result, there is a theoretical rationale for the use of antifibrinolytic agents in the treatment of postpartum haemorrhage[12,18,19].

### Potential side effects of tranexamic acid

As TXA inhibits the breakdown of fibrin deposits already formed, it might theoretically increase the risk of thromboembolism. However, the systematic review of TXA in surgery did not show statistically significant increases in the risks of any of the thromboembolic events assessed (Table 1)[20].

**Table 1:** Effect of TXA

Events	RR	95% CI
Myocardial infarction	0.96	0.48-1.90
Stroke	1.25	0.47-3.31
Deep venous thrombosis	0.77	0.37-1.61
Renal failure	0.73	0.16-3.32

During pregnancy, women have an increased risk of thromboembolic events, compared with non-pregnant women. The absolute risk of symptomatic venous thrombosis during pregnancy has been estimated to be between 0.5 and 3.0 per 1,000 women based on studies using radiographic documentation [21-23]. Studies using objective criteria for diagnosis have found that ante-partum deep vein thrombosis (DVT) is as common as postpartum thrombosis and occurs with equal frequency in all three trimesters[21].

A population-based cohort study estimated an incidence of thromboembolic events to be 200 per 100,000 woman-years[24]. DVT was three times more common than pulmonary embolism and thromboembolic events were five times more likely in the postpartum period than during the pregnancy. This was particularly evident with pulmonary embolism which was 15 times more likely to occur in the postpartum period than during the pregnancy. Thromboembolic events will be collected routinely as part of the data collection process for this trial.

TXA passes into breast milk in very low concentrations, approximately one hundredth of the concentration in the maternal blood. An antifibrinolytic effect in the infant is very unlikely at this low concentration[25]. The thromboembolic effects on breastfed babies will be assessed in this trial.

TXA is not a new drug and is generally well tolerated. Adverse events are uncommon and usually manifest as nausea or diarrhoea, or occasionally as orthostatic reactions[12].

### Objective

The WOMAN trial will provide reliable evidence as to whether the antifibrinolytic agent tranexamic acid reduces mortality, hysterectomy and other morbidities in women with clinically diagnosed postpartum haemorrhage. Thromboembolic effects on breastfed babies will be assessed.

## Methods, design, discussion

### Overview

This trial is a large, pragmatic, randomised, double blind, placebo controlled trial to quantify the effects of the early administration of tranexamic acid on death, hysterectomy and other relevant outcomes. 15,000 adult women, who have clinically diagnosed postpartum haemorrhage and who fulfil the eligibility criteria, will be randomised to receive either TXA or placebo. The eligibility criteria are based on the uncertainty principle.

#### Pragmatic design and the uncertainty principle

The pragmatic design will allow us to find out how effective the treatment actually is in routine everyday practice. The eligibility criteria are based on the uncertainty principle. This approach to trial eligibility is well established[26]. A patient can be enrolled if, and only if, the responsible clinician is substantially uncertain as to which of the trial treatments would be most appropriate for that particular woman (see graph 1). A woman should not be enrolled if the responsible clinician or the woman (or her representative) are for any medical or non-medical reasons reasonably certain that one of the treatments that might be allocated would be inappropriate for this particular individual (in comparison with either no treatment or some other treatment that could be offered to the patient in or outside the trial). Using the uncertainty principle should allow the process of this trial to be closer to what is appropriate in normal medical practice. Clinicians, women and their representatives will be provided with information about the trial treatment to assist them in their judgement.

#### Randomisation

Women eligible for inclusion should be randomised, and the study treatment started, as soon as possible. The Entry form (see Additional file 1, Form 1: Entry form) will be used to assess eligibility and collect baseline information. The next consecutively numbered treatment pack, taken from a box of eight packs, should be chosen. Once a patient has been randomised, the outcome in hospital needs to be collected even if the trial treatment is interrupted or is not actually given.

#### Follow-up

No extra tests are required for the trial but a short Outcome form (see Additional file 2, Form 2: Outcome form) must be completed directly from the medical records six weeks (42 days) after randomisation or on discharge from the randomising hospital or on death (whichever occurs first). Any adverse events which become known to the investigator will be reported up to 42 days after randomisation.

### Settings

The pragmatic nature of this trial will allow for the recruitment of women from a wide variety of health care facilities. Participating hospitals or maternal health facilities will be selected from high, middle and low income countries. Eligible women may have delivered their babies at the participating hospital or may have delivered outside the participating hospital and been admitted following the delivery of a baby. There is no limit to the maximum number of women to be recruited at each site.

### Number of patients needed

Two main factors determine the number of patients needed in a trial. These are the estimated event rate and size of the treatment effect.

### Estimated event rate

Review of the literature and data from hospital reports shows that there are wide variations in mortality after PPH worldwide, varying from about 0.6% in the United Kingdom to 2.6% in South Africa and 20% in some parts of Africa. The frequency of occurrence of peripartum hysterectomies also varies, from about 0.02% in the United Kingdom to 2% in Nigeria or 14% in Congo-Brazzaville. Based on these ranges, a baseline event rate of 2.5% for mortality and 2.5% for hysterectomy might reasonably be expected.

### Sample size and size of treatment effect that should be detectable

Assuming a control group event rate of 2.5% for mortality and 2.5% for hysterectomy with 1% of women having both a hysterectomy and then dying, a study with 15,000 women would have over 90% power (two sided alpha = 5%) to detect a clinically important 25% reduction from 4% to 3% in the primary endpoint of mortality or hysterectomy. A survey of baseline event rates among hospitals that have expressed interest in taking part shows that baseline event rates of this magnitude are realistic and that higher baseline event rates might reasonably be expected. Experience from the CRASH-1 and CRASH-2 clinical trials suggests that the anticipated rates of loss to follow-up (less than 1%) would not impact importantly on study power.

### Recruitment of collaborating investigators

The trial will recruit collaborating sites from all countries worldwide and will continue to add sites to ensure the sample size is achieved. Suitable collaborating sites and investigators will be assessed on the level of obstetric service they provide and their ability to conduct the trial. In advance of the trial starting at a site the Principal Investigator must agree to adhere to Good Clinical Practice Guidelines and all relevant regulations in their country. In addition, all relevant regulatory and ethics approvals will need to be in place.

### Eligibility

Immediately after delivery of the baby/ies, all usual care should be given for the prevention of PPH. Some bleeding is expected after delivery. However, if bleeding continues and a diagnosis of PPH is made, all usual treatments should be given and at the same time the assessment for inclusion in the trial should be made. It is important to consider inclusion as early as possible.

### Inclusion criteria

All legally adult women with clinically diagnosed postpartum haemorrhage following vaginal delivery of a baby or caesarean section; women may have delivered their babies at a participating hospital or outside a participating hospital, with hospital admission following delivery:

• where the responsible clinician is substantially uncertain as to whether or not to use TXA

• when consent has been given according to approved procedures

The clinical diagnosis of PPH **may **be based on any of the following:

• estimated blood loss after vaginal delivery of a baby > 500 mL **OR**

• >1000 mL from caesarean section **OR**

• estimated blood loss enough to compromise the haemodynamic status of the woman

### Exclusion criteria

• Women for whom the responsible clinician considers there is a clear indication for TXA should not be randomised.

• Women for whom the responsible clinician considers there is a clear contraindication for TXA should not be randomised (e.g. a known thromboembolic event during pregnancy).

The fundamental eligibility criterion is the responsible clinician's 'uncertainty' as to whether or not to use an antifibrinolytic agent in a particular woman with postpartum haemorrhage.

**Figure 1:**
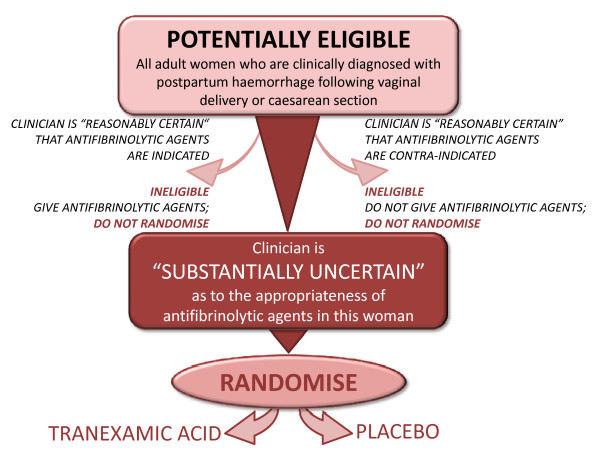
**Eligibility graph**.

The TXA summary of product characteristics [25] and an Investigator's Brochure will be provided to investigators to ensure they have adequate information when considering the risk-benefit and the appropriateness of the trial for each woman (Figure 1).

### Consent and ethical considerations

This trial will be carried out worldwide and will include women soon after delivery of a baby. Postpartum haemorrhage is an emergency situation and clinical activities will be directed towards the provision of emergency care. Eligible women have a life threatening condition. Furthermore, their physical, mental and emotional state may be altered as a result of their blood loss or labour pains or by drugs administered during the labour. The consent process in this situation requires careful consideration bearing in mind applicable regulatory requirements, adherence to ICH-GCP and the requirements in the Declaration of Helsinki.

#### Advance Information

The majority of women deliver without complications and it would not be in the best interest of all pregnant women to cause undue concern by providing detailed information about this trial in the antenatal/delivery period. Also, it is not possible to identify in advance those women who will go on to develop PPH, and obtain advance consent. Therefore, where possible, a summary of the trial information will be provided to pregnant women (see Additional file 3, Form 3: Brief antenatal information leaflet). Refusal to be considered for participation will be documented in the woman's medical records and her decision respected. Following delivery of her baby, and once a woman has been diagnosed with PPH, a critical clinical emergency situation exists. The risk of death is highest early after delivery. The process by which information will be given and consent obtained will depend on the need for urgent clinical intervention and her physical, mental and emotional state. Also, the availability and ability of a personal representative to make a decision on the woman's behalf will have to be taken into consideration. The approach which will allow the woman to have the most input into the decision making process without endangering her life will be utilised:

#### a) The woman is fully competent

The woman will be approached with the agreement of the primary carer (the midwife or doctor) at the time of diagnosis. Factors which may impair her decision making process including pain, altered level of consciousness due to drugs given and degree of blood loss, will be taken into consideration. An Information Sheet (see Additional file 4, Form 4: Information sheet for the woman and her representative) will be provided and the study will be discussed with her and a written consent obtained (see Additional file 5, Form 5: Patient consent form). If the woman is unable to read or write, then the information sheet may be read to her and she may then mark the consent form with either a cross or thumbprint. In this event, a witness NOT associated with the trial, must provide a full signature confirming the mark.

#### b) The woman's mental capacity is impaired and either a Personal or Professional representative is available

Information should be given to the woman taking her level of mental impairment into consideration. Oral refusal by the woman should be respected and she should not be enrolled.

(1) If a Personal Representative (PeR) who is knowledgeable about the woman's values and beliefs is available, an Information Sheet will be provided. Opportunity for questions should be given and written consent obtained. If the PeR is unable to read or write, then the information sheet may be read to him/her and a mark with either a cross or thumbprint made on the consent form (see Additional file 6, Form 6: Representative consent form). In this event, a witness NOT associated with the trial, must provide a full signature confirming the mark.

(2) If a Personal Representative is not available and the woman is unable to provide valid informed consent, then an independent doctor/midwife/other site staff allowed to fulfil this role (ideally the primary carer if s/he is not part of the trial team) may be asked to consent as a Professional Representative (PrR). Informed consent given by a representative shall represent the woman's presumed will.

(3) The woman's mental capacity is impaired and neither a Personal nor Professional representative is available: In situations where the woman is facing a clinical emergency and no PeR/PrR is available, the investigator and ONE independent person (doctor or midwife) who is not participating in this trial may enrol the woman into the trial by certifying in writing in the woman's medical records that:

• the woman is facing a life-threatening postpartum haemorrhage;

• the woman is unable to give her consent as a result of her medical condition;

• it is not feasible to contact the woman's PeR/PrR to obtain consent within the window period; and

• neither the woman nor the woman's PeR/PrR nor any member of the family has informed the investigator of any objections to the woman being used as a participant in this trial.

**Figure 2:**
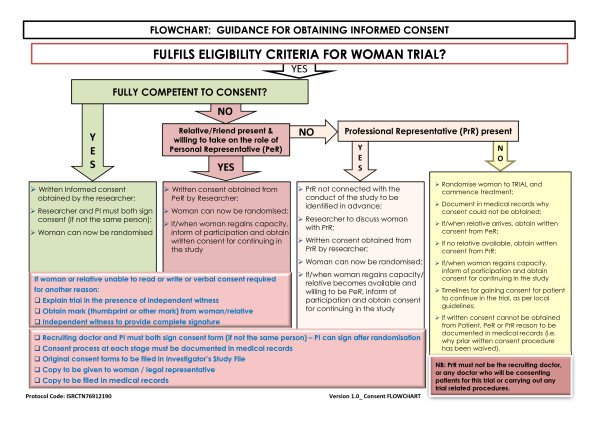
**Consent procedure diagram**.

For women enrolled under such emergency consent procedure, the woman or her PeR or PrR should be informed about the trial as soon as it is possible and asked to consent for continuation of any trial procedure. The requirements of the relevant ethics committee will be adhered to at all times (Figure 2).

### Randomisation

Randomisation codes will be generated and secured by an independent statistical consultant from Sealed Envelope Ltd (UK). The codes will be made available to Brecon Pharmaceuticals Limited (UK) explicitly for the treatment packs to be created in accordance with the randomisation list. Eligibility will be determined from the routinely collected clinical information and no trial-specific tests are required. Women eligible for inclusion should be randomised to receive either active (tranexamic acid) or placebo (sodium chloride 0.9%) treatment and the trial treatment started as soon as possible.

Baseline information will be collected on the trial entry form and the next lowest consecutively numbered pack will be taken from a box of eight treatment packs. When the treatment ampoule is confirmed as being intact, at this point the patient is considered to be randomised onto the trial. The entry form data will be sent to the Trial Coordinating Centre as soon as possible. Once a patient has been randomised, the outcome of the woman should be obtained even if the trial treatment is interrupted or is not actually given.

### Treatment

Tranexamic acid will be compared with matching placebo (sodium chloride 0.9%).

### Dose selection

In randomised trials of antifibrinolytic agents in surgery, TXA dose regimens vary widely. Loading doses range from 2.5 mg/kg to 100 mg/kg and maintenance doses from 0.25 mg/kg/hour to 4 mg/kg/hour given over periods of one to twelve hours. Studies examining the impact of different doses of tranexamic acid on bleeding and transfusion requirements showed no significant differences between a high dose and a low dose. Studies in cardiac surgery have shown that a 10 mg/kg initial dose of TXA followed by an infusion of 1 mg/kg/hour produces plasma concentrations sufficient to inhibit fibrinolysis in vitro. Horrow et al (1995) examined the dose-response relationship of TXA and concluded that 10 mg/kg followed by 1 mg/kg/hour decreases bleeding in cardiac surgery, but larger doses did not provide any additional haemostatic benefit[27]. Trials of the use of TXA for the prevention of obstetric haemorrhage used TXA at a dose of 1 gram without major complications[14]. In the emergency situation, the administration of a fixed dose is more practicable since weighing women with PPH would be difficult. Therefore, a fixed dose of 1 gram of TXA initially followed by 1 gram if bleeding continues, which is within the dose range which has been shown to inhibit fibrinolysis and provide haemostatic benefit, has been selected for the WOMAN trial. On the basis of experience in surgery, the dose selected would be efficacious for larger patients (>100 kg) but also safe in smaller patients (<50 kg), as the estimated dose/kg that the patients in the latter group would receive has been applied in other trials without significant adverse effects.

### Drug manufacture, blinding and supply of trial treatment

The active trial drug tranexamic acid (Cyklokapron^® ^Injection) will be purchased on the open market in the UK. TXA is manufactured by Pfizer Ltd under Marketing Authorisation Number: PL 00032/0314. The Marketing Authorisation guarantees that the product has been manufactured and released in accordance with the United Kingdom's Good Manufacturing Regulations.

Placebo (sodium chloride 0.9%) will be manufactured specially to match the tranexamic acid by South Devon Healthcare NHS Trust, Kemmings Close, Paignton, Devon, TQ4 7TW, under UK Manufacturer's authorisation Number: MS13079/MA(IMP) 13079.

Ampoules and packaging will be identical in appearance. The blinding process and first stage Qualified Person (QP) release will be done by Brecon Pharmaceuticals Limited, Wye Valley Business Park, Hay-on-Wye, Hereford HR3 5PG, under UK Manufacturer's authorisation Number MIA 11724/MIA IMP 11724. The blinding process will involve complete removal of the original manufacturer's label and replacement with the clinical trial label bearing the randomisation number which will be used as the pack identification. Other pack label text will be identical for both TXA and placebo treatments and will be in compliance with requirements for investigational medicinal products. Treatment packs containing TXA and placebo will be packed in balanced blocks of 8 (4 TXA:4 Placebo) into a box in random order. Brecon Pharmaceuticals Limited will also be responsible for maintaining the Product Specification File (PSF) until final database lock and unblinding of the trial data. Quality control checks to assure blinding process will be performed on a random sample of final QP released drug packs. High Performance Liquid Chromatography analyses (HPLC) separation of known tranexamic acid will be assessed against blinded samples to confirm which ampoule contains the placebo and active treatments. The tested samples will be unblinded to assure accuracy of blinding. The Trials Coordinating Centre (TCC) will be responsible for assuring all relevant approvals are available at the TCC before release of the trial treatment to a site.

### Administration of trial treatment

Each treatment pack will contain:

• 4 × 500 mg ampoules of tranexamic acid or placebo

• 2 × sterile 10 mL syringe and 21FG needle

• Stickers (for attaching to data forms and patient medical records)

#### Dose 1

2 ampoules = 1 gram - to be administered by intravenous injection at an approximate rate of 1 mL/minute to all randomised women as soon as possible after randomisation.

#### Dose 2

2 ampoules = 1 gram - If after 30 minutes bleeding continues, or if it stops and restarts within the 24 hours after the first dose, a second dose may be given. To be administered by intravenous injection at an approximate rate of 1 mL/minute. The trial treatment injections should not be mixed with blood for transfusion, or infusion solutions containing penicillin or mannitol.

### Other treatments for postpartum haemorrhage

There is a wide spectrum of first and second line treatments of postpartum haemorrhage. As the trial will be conducted worldwide, each participating site should follow its own clinical guidelines for the treatment of postpartum haemorrhage. Information on other treatments given will be collected on the outcome form. Tranexamic acid or placebo would be an additional treatment to the routine management of postpartum haemorrhage.

### Adverse events (AE)

TXA has a well documented safety profile. No increase in thromboembolic risks associated with its use has been shown to date. However, as discussed in Section 1.3 an expected complication of pregnancy is an increased risk of thromboembolic events. This trial will collect data on all thromboembolic events as secondary outcomes, and all such events are routinely reported to the independent data monitoring committee (DMC) for unblinded review.

### Definitions

#### Adverse event (AE)

Any untoward medical occurrence affecting a trial participant during the course of a clinical trial.

#### Serious Adverse Event (SAE)

A serious adverse event (experience) is any untoward medical occurrence that at any dose results in death; is life-threatening; requires inpatient hospitalisation or prolongation of existing hospitalisation; results in persistent or significant disability/incapacity; or is a congenital anomaly/birth defect.

#### Adverse Reaction (AR)

An adverse event when there is at least a possibility that it is causally linked to a trial drug or intervention.

#### Serious Adverse Reaction (SAR)

SAE that is thought to be causally linked to a trial drug or intervention.

#### Suspected Unexpected Serious Adverse Reaction (SUSAR)

An *unexpected *occurrence of a SAR; there need only be an index of suspicion that the event is a previously unreported reaction to a trial drug or a previously reported but exaggerated or unexpectedly frequent adverse drug reaction.

#### Reporting of Adverse Events for this trial

Death, life-threatening complications and prolonged hospital stay are pre-specified outcomes to be reported in this trial and also to the independent data monitoring committee. This clinical trial is being conducted in a critical emergency condition, using a drug in common use. It is important to consider the natural history of the critical medical event affecting each woman enrolled, the expected complications of this event and the relevance of the complications to TXA.

Adverse events to be reported using an adverse event reporting form will be limited to those NOT already listed as primary or secondary outcomes, yet, which might reasonably occur as a consequence of the trial drug. Events that are part of the natural history of the primary event of PPH or expected complications of PPH should not be reported as adverse events.

In addition, if a woman is discharged from the randomising hospital before day 42 and is readmitted to hospital, requires medical care for any reason or is known to have died, an 'adverse event form' should be completed irrespective of the cause. If a Serious Adverse Event occurs, this should be logged by calling the Trial Coordinating Centre Emergency Helpline and a written report submitted within 24 hours. The TCC will coordinate the reporting of all SAEs to all relevant Regulatory Agencies, Ethics Committees and local investigators as per local legal requirements.

### Unblinding

In general there should be no need to unblind the allocated treatment. If some contraindication to antifibrinolytic therapy develops after randomisation, e.g. clinical evidence of thrombosis, the trial treatment should simply be stopped and all usual standard care given. Unblinding should be done only in those rare cases when the clinician believes that clinical management depends importantly upon knowledge of whether the patient received antifibrinolytic or placebo. In those few cases when urgent unblinding is considered necessary, a 24-hour telephone service will be available and details provided in the Investigator's Study File and wall posters. The caller will be told whether the patient received antifibrinolytic or placebo. An unblinding report form should be completed by the investigator.

### Measures of outcome

After a patient has been randomised, outcome in hospital will be collected even if the trial treatment is interrupted or is not actually given. No extra tests are required but a short single page Outcome Form will be completed 6 weeks (42 days) after randomisation, at discharge from the randomising hospital or at death (whichever occurs first).

#### Primary Outcome

The primary outcome is the proportion of women who die or undergo hysterectomy. The primary cause of death will be described.

#### Secondary outcomes

(a) Death.

(b) Surgical Interventions including hysterectomy; brace suture (B-Lynch/Cho); selective arterial embolisation; laparotomy for other reasons; manual removal of placenta; intrauterine tamponade (packing or gauzing the uterine cavity, condom-catheter, any other method of intrauterine tamponade); artery ligation, to achieve haemostasis.

(c) Blood transfusion - blood or blood component units transfused.

(d) Health Related Quality of life (HRQoL) will be measured by the proxy version of the EQ-5D at discharge from the randomising hospital or in hospital at 42 days after randomisation. The EQ-5D includes single item measures of mobility, self-care, usual activities, pain/discomfort and anxiety/depression. Each item is coded using 3 levels (1 = no problems; 2 = some problems; 3 = severe problems). The instrument includes a global rating of current health using a visual analogue scale (VAS) ranging from 0 (worst imaginable) to 100 (best imaginable). The EQ-5D is a generic measure of health status that provides a simple descriptive profile and a single index value that can be used in the clinical and economic evaluation of health care.

(e) Thromboembolic events (myocardial infarction, strokes, pulmonary embolism, deep vein thrombosis).

(f) Medical events including renal failure, Adult Respiratory Distress Syndrome, hypertensive disorders of pregnancy (including HELLP Syndrome, eclampsia, toxaemia of pregnancy) and other adverse events reported.

(g) Length of stay at hospital/time spent at an intensive care unit.

(h) Receipt of mechanical ventilation.

(i) Status of baby/ies: The health status of the baby/ies will be ascertained and information collected on any thromboembolic events in breastfed babies.

(j) Cost-effectiveness analysis: An economic analysis will be relevant if TXA clearly demonstrates efficacy in achieving its clinical aims. In this case, the study will be undertaken in the form of a cost-effectiveness analysis with the aim of estimating the incremental cost-effectiveness ratio comparing the use of TXA with normal clinical practice. Analysis will be based on adjusted life years gained. A further analysis will explore the use of the EQ-5D data to quality adjust survival. In this study, the economic analysis is clearly bounded as virtually all significant resource use will occur in the initial period of hospitalisation. As such, neither a long-term resource analysis nor an analysis of out of hospital costs will be required. The trial use of TXA is likely to mirror its use in normal clinical practice, hence the cost-effectiveness estimated in the trial (adjusted for protocol driven costs) will closely approximate cost-effectiveness in actual clinical practice. Data on physical resource consumption (e.g. length and nature of hospital stay) will be collected for each patient and a common unit cost at a country level will be applied. A sensitivity analysis will be undertaken to assess the robustness of the economic analysis in response to variations in key variables such as drug prices. In all cases, the economic analysis will be integrated with the clinical trial procedures to optimise efficiency and minimise inconvenience to patients.

### Data collection

This trial will be coordinated from LSHTM and conducted in hospitals in low, middle and high income countries. Most recruitment will be in countries with high rates of mortality and morbidity from postpartum haemorrhage. Data will be collected at each site by local investigators and transmitted to the TCC. Only data outlined on the entry, outcome and adverse event forms will be collected for this trial.

Relevant data on an entry form will be collected before randomisation to assess eligibility and the form completed if randomised. The outcome form should be completed at death, discharge from the randomising hospital or 6 weeks (42 days) after randomisation whichever occurs first. This data should be collected from the routine medical records of the woman and her baby/ies as no special tests are required.

If the woman (or her PeR or PrR) withdraws a previously given informed consent or refuses to consent for continuation in the trial, or if the woman dies and no consent is available from either a PeR/PrR, her data will be handled as follows:

• Data collected to the point of withdrawal of consent will be used as part of the intention to treat analysis.

• All relevant adverse events identified will be reported as required to all relevant authorities.

To allow for variation in available technology for data transfer a variety of methods will be used in this trial. Data will be collected by the investigator on paper case report forms (CRFs) and transmitted to the TCC either as a paper form (by fax or email) or by entering the data directly into the trial database. Data can also be transmitted by entry onto electronic data files which can be emailed or uploaded to the TCC secure web server. In cases where electronic data files are used, data stored on the investigator's computer(s) and data during transfer will be secured by encryption. The data will be used in accordance with local law and ethics committee approval.

### Monitoring

GCP section 5.18.3 states in regard to monitoring, *"The determination of the extent and nature of monitoring should be based on considerations such as the objective, purpose, design, complexity, blinding, size and endpoints of the trial. In general there is a need for on-site monitoring, before, during, and after the trial; however in exceptional circumstances the sponsor may determine that central monitoring in conjunction with procedures such as investigators training and meetings, and extensive written guidance can assure appropriate conduct of the trial in accordance with GCP. Statistically controlled sampling may be an acceptable method for selecting the data to be verified."*

This trial is a large, pragmatic, randomised placebo controlled trial. The intervention (tranexamic acid) has marketing authorisation in many countries and has been in clinical use for over 40 years. Its safety profile is well established and no significant serious adverse events associated with its use have been identified. The trial will routinely collect data on adverse events which may theoretically be associated with this product and the condition under investigation, and these will be reviewed routinely by the independent Data Monitoring Committee (DMC). Other than consent, the administration of the trial drug using a routine clinical procedure and collecting routine clinical information from the medical records, there are no complex procedures or interventions for the participants or investigators in this trial. Clinical management for underlying conditions will remain as per each hospital's standard protocol. Based on these factors, the probability of harm or injury (physical, psychological, social or economic) occurring as a result of participation in this research study has been assessed as low risk to participants in each of these categories. Based on the low risks associated with this trial, a Monitoring Plan to assure appropriate conduct of the trial will be developed which will incorporate 100% central monitoring in conjunction with procedures such as investigator training and meetings and written guidance. In addition, all data will be subject to statistical monitoring and at least 10% of data will be subjected to on-site monitoring. Investigators/institutions are required to provide direct access to source data/documents for trial-related monitoring, audits, ethics committee review and regulatory inspection. All trial related and source documents must be kept for five years after the end of the trial.

### End of trial for participants

The trial ends either at death, discharge, or six weeks post-randomisation, whichever occurs first. If during the treatment phase a woman develops an adverse event the trial drug should be stopped, woman treated in line with local procedures and then followed up.

The trial may be terminated early by the Trial Steering Committee (TSC). The DMC may give advice/recommendation for the early termination of the trial but the TSC is responsible for the final decision.

### Analysis

The main analyses will compare all those allocated antifibrinolytic treatment versus those allocated placebo, on an 'intention to treat' basis, irrespective of whether they received the allocated treatment or not. Results will be presented as appropriate effect estimates with a measure of precision (95% confidence intervals). Subgroup analyses for the primary outcome will be based on type of delivery (vaginal or caesarean section); administration or not of prophylactic uterotonics; and on whether the clinical decision to consider trial entry was based primarily on estimated blood loss alone or on haemodynamic instability. Interaction test will be used to test whether the effect of treatment (if any) differs across these subgroups. Between-sites heterogeneity in effectiveness will be explored. All analyses will be conducted in STATA. A detailed Statistical Analysis Plan setting out full details of the proposed analyses will be finalised before the trial database is locked for final analysis.

### Sponsorship and trial management

The WOMAN Trial is sponsored by the London School of Hygiene & Tropical Medicine (LSHTM) and its responsibilities coordinated by the Trial Coordinating Centre (TCC). The TCC may delegate responsibilities to third parties which will be outlined in relevant agreements. The responsibilities of the TCC will be overseen by the Trial Management Group.

### Indemnity

LSHTM accepts responsibility attached to its sponsorship of the trial and, as such, would be responsible for claims for any non-negligent harm suffered by anyone as a result of participating in this trial. The indemnity is renewed on an annual basis and LSHTM assures that it will continue renewal of the indemnity for the duration of this trial.

### Protocol development

The Protocol Committee consists of the following investigators who will be responsible for the development of and agreeing to the final protocol. Subsequent changes to the final Protocol will require the agreement of the Trial Steering Committee.

• Chief investigator: Professor Ian Roberts

• Clinical experts: Professor Zarko Alfirevic, Dr Metin Gülmezoglu, Professor Carine Ronsmans

• Trial management: Ms Haleema Shakur

• Statistician: Professor Diana Elbourne

### Independent data monitoring committee (DMC)

• Professor Sir Iain Chalmers, James Lind Initiative, Oxford, UK (Large scale randomised controlled trials; Obstetric care).

• Professor Pisake Lumbiganon, Professor of Obstetrics & Gynaecology; Convenor, Thai Cochrane Network; Faculty of Medicine, Khon Kaen University, Thailand (Obstetric care).

• Dr Gilda Piaggio, Statistika Consultoria, São Paulo, Brazil (Statistician, extensive experience of reproductive health and research at the World Health Organization).

Mortality and severe morbidity is expected within the target population. To provide protection for study participants, an independent DMC has been appointed for this trial to oversee the safety monitoring. The DMC will review on a regular basis accumulating data from the ongoing trial and advise the Trial Steering Committee regarding the continuing safety of current participants and those yet to be recruited, as well as reviewing the validity and scientific merit of the trial.

The DMC composition, name, title and address of the chairman and of each member, will be given in the DMC Charter which will be in line with that proposed by the DAMOCLES Study Group[28]. Membership includes expertise in the relevant field of study, statistics and research study design. The DMC Charter includes, but is not limited to, defining:

(a) the schedule and format of the DMC meetings

(b) the format for presentation of data

(c) the method and timing of providing interim reports

(d) stopping rules

#### Standard Operating Procedures

The Data Monitoring Committee (DMC) has the responsibility for deciding whether, while randomisation is in progress, the unblinded results (or the unblinded results for a particular subgroup), should be revealed to the TSC. The DMC Charter states that they will do this if, and only if, two conditions are satisfied:

(1) the results provide proof beyond reasonable doubt that treatment is on balance either definitely harmful or definitely favourable for all, or for a particular category of, participants in terms of the major outcome; (2) The results, if revealed, would be expected to substantially change the prescribing patterns of clinicians who are already familiar with any other trial results that exist. Exact criteria for "proof beyond reasonable doubt" are not, and cannot be, specified by a purely mathematical stopping rule, but they are strongly influenced by such rules. DMC Charter is in agreement with the Peto-Haybittle [29,30] stopping rule whereby an interim analysis of major endpoint would generally need to involve a difference between treatment and control of at least three standard errors to justify premature disclosure. An interim subgroup analysis would, of course, have to be even more extreme to justify disclosure. This rule has the advantage that the exact number and timing of interim analyses need not be pre-specified. In summary, the stopping rules require extreme differences to justify premature disclosure and involve an appropriate combination of mathematical stopping rules and scientific judgment.

### Trial steering committee (TSC)

• Professor Adrian Grant (Chair), Director, Health Services Research Unit, University of Aberdeen (Health Services Research; Randomised Control Trials).

• Professor Ian Roberts, London School of Hygiene & Tropical Medicine (Epidemiology; Randomised Control Trials; Conduct of large scale international trials).

• Dr Metin Gülmezoglu, World Health Organization, Geneva (Obstetrician; Coordinating Editor of the WHO Reproductive Health Library; Randomised Control Trials).

• Dr Kaosar Afsana, BRAC Health Programme, Bangladesh (Reproductive & Sexual Health & Rights; Rural and Urban Maternal, Neonatal and Child Health Programme in BRAC).

• Dr Oladapo Olayemi, University College Hospital, Ibadan, Nigeria (Consultant Obstetrician; perspective on obstetrics in a developing country).

• Professor Beverley Hunt, Kings College, London (Professor of Thrombosis & Haemostasis, Randomised Control Trials).

The role of the TSC is to provide overall supervision of the trial. In particular, the TSC will concentrate on the progress of the trial, adherence to the protocol, patient safety and consideration of new information. The TSC must be in agreement with the final Protocol and, throughout the trial, will take responsibility for:

(a) major decisions such as a need to change the protocol for any reason

(b) monitoring and supervising the progress of the trial

(c) reviewing relevant information from other sources

(d) considering recommendations from the DMC

(e) informing and advising the Trial Management Group on all aspects of the trial The steering committee consists of experienced obstetric experts, clinical trialists as well as a Reproductive & Sexual Health & Rights representative. Face to face meetings will be held at regular intervals determined by need, but no less than once a year. A TSC Charter will be agreed at the first meeting which will detail how it will conduct its business.

When outcome data are available for 1,000 trial participants, the TSC will review the rate of recruitment into the trial and the overall event rates. The TSC will consider the extent to which the rate of recruitment and the event rates correspond to those anticipated before the trial and will take whatever action is needed in light of this information.

### Collaborators' responsibilities

Coordination within each participating hospital will be through a local Principal Investigator whose responsibility will be detailed in an agreement in advance of starting the trial and will include:

• Ensure all necessary approvals are in place prior to starting the trial

• Delegate trial related responsibilities only to suitably trained and qualified personnel

• Train relevant medical and nursing staff who see obstetric patients and ensure that they remain aware of the state of the current knowledge, the trial and its procedures (there are wall charts, pocket summaries and a set of slides to assist with this)

• Agree to comply with the final trial protocol and any relevant amendments

• Ensure that all women with postpartum haemorrhage are considered promptly for the trial

• Ensure consent is obtained in line with local approved procedures

• Ensure that the patient entry and outcome data are completed and transmitted to the TCC in a timely manner

• Ensure the Investigator's Study File is up-to-date and complete

• Ensure all Adverse Events are reported promptly to the TCC

• Accountability for trial treatments at their site

• Ensure the trial is conducted in accordance with ICH GCP and fulfils all national and local regulatory requirements

• Allow access to source data for monitoring, audit and inspection

• Be responsible for archiving all original trial documents including the data forms for five years after the end of the trial

### Trial management group (TMG) and trial coordinating centre (TCC) responsibilities

The Trial Management Group will consist of the Protocol Committee members (Section 3.3) plus a trial manager, data manager and trial administrator. The TCC will act on behalf of the Sponsor and will be responsible to the TMG to ensure that all Sponsor's responsibilities are carried out. The responsibilities will include (but not limited to):

• Report to the Trial Steering Committee

• Maintain the Trial Master File

• Identify trial sites

• Confirm all approvals are in place before release of the trial treatment and the start of the trial at a site

• Provide training about the trial

• Provide study materials

• Data management centre

• 24-hour advice and unblinding service

• Give collaborators regular information about the progress of the study

• Respond to any questions (e.g. from collaborators) about the trial

• Ensure data security and quality and observe data protection laws

• Safety reporting

• Ensure trial is conducted in accordance with the ICH GCP

• Statistical analysis

• Publication of trial results

### Contacting the TCC in an emergency

For urgent enquiries, adverse event reporting and unblinding queries investigators can contact the 24-hour telephone service provided by the TCC. A central telephone number is given in the Investigator's Study File and posters.

### Publication and dissemination of results

All efforts will be made to ensure that the trial protocol and results arising from the WOMAN trial are published in an established peer-reviewed journal. At least one publication of the main trial results will be made. All publications will follow relevant external guidance such as the *'Uniform Requirements for Submission of Manuscripts to Biomedical Journals' *issued by the International Committee of Medical Journal Editors (ICMJE) (2008 update) and the CONSORT statement [31,32]. Links to the publication will be provided in all applicable trial registers. Dissemination of results to patients will take place via the media, trial website http://www.womantrial.lshtm.ac.uk/ and relevant patient organisations. Collaborating investigators will play a vital role in disseminating the results to colleagues and patients.

The success of the trial will be dependent entirely upon the collaboration of midwives, nurses and doctors in the participating hospitals and those who hold key responsibility for the trial. Hence, the credit for the study will be assigned to the key collaborator(s) from a participating site as it is crucial that those taking credit for the work have actually carried it out. The results of the trial will be reported first to trial collaborators.

### Financial support

LSHTM is funding the run-in costs for this trial and up to 2,000 patients' recruitment. Full funding is being sought from public funding organisations. Funding for this trial covers meetings and central organisational costs only. Pfizer, the manufacturer of tranexamic acid, have provided the funding for the trial drug and placebo used for this trial. The design and management of the study are entirely independent of the manufacturers of tranexamic acid, which is not a new product.

Large trials of such drugs, involving many hospitals, are important for future patients, but are practicable only if those collaborating in them do so without payment (except for recompense of any minor local costs that may arise). Agreement for repayment of local costs will be made in advance.

## Abbreviations

AE: Adverse Event; AR: Adverse Reaction; CONSORT: CONsolidated Standards of Reporting Trials; CRF: Case Report Form; DMC: Data Monitoring Committee; DVT: Deep Vein Thrombosis; FG: French Gauge; GCP: Good Clinical Practice; HELLP: Hemolytic anemia, Elevated Liver enzymes and Low Platelet count; HPLC: High Performance Liquid Chromatography; HRQoL: Health Related Quality of Life; ICH GCP: International Conference on Harmonisation of Good Clinical Practice; ICMJE: International Committee for Medical Journal Editors; Kg: Kilogram; LSHTM: London School of Hygiene & Tropical Medicine; MDG: Millennium Development Goal; Mg: Milligram; mL: Millilitre; PeR: Personal Representative; PPH: Postpartum Haemorrhage; PrR: Professional Representative; PSF: Product Specification File; QP: Qualified person; SAE: Serious Adverse Event; SAR: Serious Adverse Reaction; SUSAR: Suspected Unexpected Serious Adverse Reaction; TCC: Trials Coordinating Centre; TMG: Trial Management Group; TSC: Trial Steering Committee; TXA: Tranexamic Acid; UK: United Kingdom; VAS: Visual Analogue Score; WHO: World Health Organization.

## Competing interests

The authors declare that they have no competing interests. Funding has been received from Pfizer Inc. as an unrestricted educational grant for the production of the trial treatment.

## Authors' contributions

Haleema Shakur, Diana Elbourne, Ian Roberts, Metin Gülmezoglu, Zarko Alfirevic, and Carine Ronsmans have made substantial contributions to the concept and design of the trial. They were all involved in drafting the protocol, revising it critically for important intellectual content and have given approval of the final version to be used in this trial. Elizabeth Allen contributed to the statistical analysis plan for the trial protocol.

## Supplementary Material

Additional file 1 **Form 1**. Entry form, pages 1 and 2. Click here for file

Additional file 2 **Form 2**. Outcome form, pages 1 and 2. Click here for file

Additional file 3 **Form 3**. Brief advance information leaflet, pages 1 and 2. Click here for file

Additional file 4 **Form 4**. Information sheet for the woman and her representative, pages 1-3. Click here for file

Additional file 5 **Form 5**. Patient consent form. Click here for file

Additional file 6 **Form 6**. Representative consent form. Click here for file
