# Supplementary material for: The WOMAN Trial (World Maternal Antifibrinolytic Trial): tranexamic acid for the treatment of postpartum haemorrhage: an international randomised, double blind placebo controlled trial
Source: Trials. 2010 Apr 16;11:40. doi: 10.1186/1745-6215-11-40 (PMC2864262; doi:10.1186/1745-6215-11-40)
Supplement: Additional file 1 — Form 1. Entry form, pages 1 and 2. [file 1745-6215-11-40-S1.PDF]

woman
World Maternal Antifibrinolytic Trial

ENTRY FORM

PLEASE COMPLETE BEFORE RANDOMISING THE WOMAN

ABOUT YOUR HOSPITAL *(please ensure all information below is contained in the medical records)*

**1.** Country	
**2.** Hospital code *(in your Study File)*	

ABOUT THE PATIENT

**3.** Patient's initials *(first name/last name)*		**4.** Patient Hospital identification number				
**5.** Do you know her date of birth? *(circle)*	a) YES	*day*	*month*	*year*	b) NO – approximate age	*years*

ABOUT THE DELIVERY

**6.** Delivery of the baby in this hospital? *(circle)*	YES	NO			
**7.** Type of delivery *(circle)*	VAGINAL	CAESAREAN SECTION			
**8.** Date of delivery	*day*	*month*	*year*		
**9.** Time of delivery *(24-hour clock)*	*hours*	*minutes*			
**10.** Placenta fully delivered? *(circle)*	YES	NO			
**11.** Primary cause of haemorrhage? *(circle)*	UTERINE ATONY	PLACENTA PRAEVIA/ACCRETA	SURGICAL TRAUMA/TEARS	OTHER	UNKNOWN
**12.** Systolic Blood Pressure		*mmHg (most recent measurement prior to randomisation)*			
**13.** Estimated volume of blood loss		*Millilitres (estimated from delivery of baby to immediately prior to randomisation)*			
**14.** Any uterotonic prophylaxis given?	YES	NO	UNKNOWN		
**15.** Clinical signs of haemodynamic instability?	YES	NO	*Haemodynamic instability assessment based on clinical signs (e.g.low BP, tachycardia, falling urine output) that requires an intervention (e.g. intravenous fluids)*		

RANDOMISATION INFORMATION

**16.** Eligible? *(circle)*	**YES** *(Get the lowest available number treatment pack and follow instructions)*		**NO** *(Do not randomise, record on screening log)*	
**17.** Consent obtained from? *(circle)*	Woman	Relative	Other representative	Waiver

18. Insert treatment pack number here **BOX** ☐☐☐☐ **PACK** ☐☐

**19.** Date of randomisation	*day*	*month*	*year*	**20.** Time of randomisation *(24-hour clock)*	*hours*	*minutes*
**21.** a)Name of person randomising patient		b) Signature				

PLEASE SEND THIS FORM TO THE COORDINATING CENTRE **IMMEDIATELY** AFTER RANDOMISATION

SEE GUIDANCE OVERLEAF

DATA FORMS GUIDANCE

AFTER COMPLETING THIS PAPER FORM, YOU CAN:

- Enter these data directly into the trial database (username and password required)

  www.thewomantrial.Lshtm.ac.uk

- Complete an Electronic Data Form (EDF) and send by email or upload to the trial intranet at *www.thewomantrial.Lshtm.ac.uk*
- Send as a secure scanned document by email to *woman.data@Lshtm.ac.uk*
- Fax to +44 20 7299 4663
- Store original form in the Investigator's Study File
- **PLEASE GIVE A COPY OF THIS COMPLETED FORM TO THE PERSON RESPONSIBLE FOR COMPLETING THE OUTCOME FORM AT YOUR HOSPITAL**

**NOTES:**

FOR ADVERSE EVENTS, UNBLINDING AND OTHER URGENT ENQUIRIES PLEASE TELEPHONE +44(0)7768 707500

PLEASE NOTE: IF YOUR QUERY IS NOT URGENT PLEASE USE THE NORMAL CONTACT DETAILS IN THE INVESTIGATOR'S STUDY FILE.
