# Supplementary material for: The WOMAN Trial (World Maternal Antifibrinolytic Trial): tranexamic acid for the treatment of postpartum haemorrhage: an international randomised, double blind placebo controlled trial
Source: Trials. 2010 Apr 16;11:40. doi: 10.1186/1745-6215-11-40 (PMC2864262; doi:10.1186/1745-6215-11-40)
Supplement: Additional file 2 — Form 2. Outcome form, pages 1 and 2. [file 1745-6215-11-40-S2.PDF]

OUTCOME FORM

COMPLETE AT DISCHARGE FROM THE RANDOMISING HOSPITAL,
DEATH IN HOSPITAL OR 42 DAYS AFTER RANDOMISATION, WHICHEVER OCCURS FIRST

Attach treatment pack sticker here or write box/pack number below:

☐☐☐☐ / ☐☐

1. HOSPITAL CODE

2. PATIENT

a) Patient initials		b) Patient hospital ID number			
c) Date of birth	DAY (DD)	MONTH (MM)	YEAR (YYYY)	d) If not known, estimated age	

3. OUTCOME

3.1 DEATH IN HOSPITAL

a) Date of death

DAY (DD)	MONTH (MM)	YEAR (YYYY)

b) Primary Cause of death *(tick one option)*

- ☐ Bleeding
- ☐ Pulmonary embolism
- ☐ Other – describe here:____

3.2 WOMAN ALIVE

a) Discharged home - Date of discharge

DAY (DD)	MONTH (MM)	YEAR (YYYY)

b) Transferred to another hospital - Date of transfer

DAY (DD)	MONTH (MM)	YEAR (YYYY)

c) Still in this hospital now *(42 days after randomisation)* - Date

DAY (DD)	MONTH (MM)	YEAR (YYYY)

3.3 IF ALIVE *(at 42 days or prior discharge):*

EQ-5D Questions as follows, to be rated by the Doctor/Midwife based on their knowledge of the woman. Read instructions overleaf before completing.

a) MOBILITY
- ☐ no problems in walking about
- ☐ some problems in walking about
- ☐ confined to bed

b) SELF-CARE
- ☐ no problems with self-care
- ☐ some problems with washing or dressing
- ☐ unable to wash or dress

c) USUAL ACTIVITIES *(e.g. care for baby, work, study, housework, family or leisure activities)*
- ☐ no problems with performing usual activities
- ☐ some problems with performing usual activities
- ☐ unable to perform usual activities

d) PAIN / DISCOMFORT
- ☐ no pain or discomfort
- ☐ moderate pain or discomfort
- ☐ extreme pain or discomfort

e) ANXIETY / DEPRESSION
- ☐ not anxious or depressed
- ☐ moderately anxious or depressed
- ☐ extremely anxious or depressed

f) VALUE RECORDED ON VISUAL ANALOGUE SCALE *(see reverse)*

☐☐☐

4. MANAGEMENT

a) DAYS IN INTENSIVE CARE UNIT
(if no ICU or not admitted to ICU, write '0' here)

b) MANAGEMENT *(Circle one box on every line)*

Hysterectomy	YES	NO
Manual removal of placenta	YES	NO
Intrauterine tamponade	YES	NO
Embolisation	YES	NO
Laparotomy for other reasons	YES	NO
Brace sutures of the uterus	YES	NO
Artery ligation	YES	NO
Mechanical ventilation *(exclude use in general anaesthetic for surgery)*	YES	NO

5. COMPLICATIONS *(Circle one box on every line)*

Pulmonary embolism	YES	NO
Deep vein thrombosis	YES	NO
Stroke	YES	NO
Myocardial infarction	YES	NO
Renal failure	YES	NO
Cardiac failure	YES	NO
Respiratory failure	YES	NO
Hepatic failure	YES	NO
Sepsis	YES	NO
Seizure	YES	NO

SEE GUIDANCE NOTES ON REVERSE

6. OTHER TREATMENTS FOR PPH

a) BLOOD PRODUCTS TRANSFUSION *(transfused in 42 days) (part unit = 1unit)*	YES	NO
Units whole blood/packed cells	units	
Fresh frozen plasma	units	
Other blood products	units	
b) UTEROTONICS ADMINISTERED (after PPH diagnosed) *(Circle one box on every line)*	YES	NO
Oxytocin	YES	NO
Ergometrine	YES	NO
Misoprostol	YES	NO
Prostaglandins (injectable)	YES	NO

7. TRIAL TREATMENT

a) Dose 1 given	YES	NO
b) Dose 2 given	YES	NO

8. ABOUT THE BABY

(please complete a separate form for each baby delivered alive: Sections 1, 2 and 8 only)

a) Number of live babies from this pregnancy		b) Initials of baby on this form	

c) THIS BABY *(Circle one box on each line)*

Alive	YES	NO
Healthy	YES	NO
Any confirmed thromboembolic event?	YES	NO
Breastfed at anytime after randomisation?	YES	NO

9. PERSON COMPLETING FORM

THE PI IS RESPONSIBLE FOR ALL DATA SUBMITTED

a) Name			
b) Position			
c) Signature			
d) Date	DAY (DD)	MONTH (MM)	YEAR (YYYY)

DETAILED GUIDANCE ABOUT COMPLETING THIS FORM CAN BE FOUND IN YOUR INVESTIGATORS STUDY FILE

SECTION 3.3: EQ-5D© INSTRUCTIONS

THIS SECTION OF THE OUTCOME FORM IS TO BE COMPLETED BY A DOCTOR/MIDWIFE WHO HAS PERSONAL KNOWLEDGE OF THE WOMAN. THE RESPONSE YOU GIVE SHOULD BE *YOUR* PERSPECTIVE OF THIS WOMAN'S STATUS COMPARED TO A NORMAL WOMAN POSTPARTUM.

SECTION 3.3

Please follow the EQ-5D guidance on the right.

SECTION 8

If more than one baby delivered alive from this pregnancy, please complete Sections 1, 2 and 8 on a separate outcome form for each baby. Remember to write the randomisation number in the box on the top right hand corner of each form.

HOW TO SEND

Please see detailed guidance in your investigators study file

AFTER COMPLETING THIS PAPER FORM, YOU CAN:

- Enter these data directly into the trial database (username and password required)

  www.thewomantrial.Lshtm.ac.uk

- Complete an Electronic Data Form (EDF) and send by email or upload to the trial intranet at *www.thewomantrial.Lshtm.ac.uk*
- Send as a secure scanned document by email to *woman.data@Lshtm.ac.uk*
- Fax to +44 20 7299 4663

STORE THIS ORIGINAL FORM IN YOUR SITEFILE

Questions a–e: By placing a tick in one box in each group, please indicate which statement best describes this woman's health state today. Do not tick more than one box in each group.

Question f: To help people say how good or bad a health state is, we have drawn a scale (rather like a thermometer) below on which the best state you can imagine is marked 100 and the worst state you can imagine is marked 0.

We would like you to indicate on this scale how good or bad the woman's health is today, **in your opinion**. Please do this by drawing a line from the black text box below to whichever point on the scale indicates how good or bad you think the woman's health is today.
Record the value on the reverse.

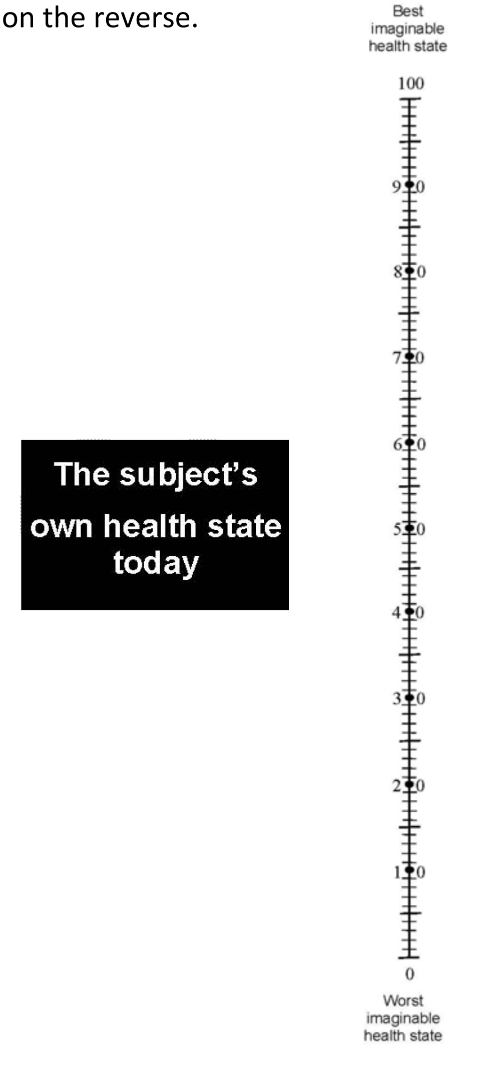

© 1990 EuroQoL Group' and 'EQ-5D™ is a trademark of the EuroQol Group
