# Supplementary material for: The WOMAN Trial (World Maternal Antifibrinolytic Trial): tranexamic acid for the treatment of postpartum haemorrhage: an international randomised, double blind placebo controlled trial
Source: Trials. 2010 Apr 16;11:40. doi: 10.1186/1745-6215-11-40 (PMC2864262; doi:10.1186/1745-6215-11-40)
Supplement: Additional file 3 — Form 3. Brief advance information leaflet, pages 1 and 2. [file 1745-6215-11-40-S3.PDF]

This hospital, like many others in this country and around the world, is involved in a research study to try and find better ways of treating women who develop severe bleeding soon after having a baby. Thank you for taking time to read this leaflet.

What is the Woman Trial about?

The WOMAN trial is a study being done to see whether using a drug called tranexamic acid will help women with severe bleeding soon after having a baby (postpartum haemorrhage) by reducing the amount of blood lost and therefore preventing them becoming too ill. This study will involve about 15,000 women worldwide. As severe bleeding is not a common problem and it is not possible to predict in advance exactly who will develop this condition, we are giving this information to all pregnant women to inform them of our plans.

What is Postpartum Haemorrhage?

Most women who give birth have no problems during or after the delivery of their baby. Following every birth there will be a small amount of bleeding from the mother – this is normal and usually nothing to worry about. However, occasionally after the baby is born there is much more bleeding. This extra bleeding is called postpartum haemorrhage (PPH). When this happens the doctors, nurses and midwives will do everything they can to stop the bleeding, because if too much blood is lost the mother may become very ill.

What causes Postpartum Haemorrhage?

Once a baby is delivered, the womb (uterus) normally continues to contract (tightening of muscles of the uterus) and this expels the placenta. After the placenta is expelled, these contractions help compress the bleeding vessels in the area where the placenta was attached. If the uterus does not contract strongly enough, these blood vessels bleed freely and haemorrhage occurs. There are many other causes of postpartum haemorrhage but this is the most common.

What is Tranexamic Acid and why use it?

Tranexamic Acid (TXA) is a drug that is used to slow down and reduce bleeding. For example, it is often used when people go for major heart surgery to stop them losing too much blood. It is also sometimes used for women who have very heavy periods. Because TXA is known to reduce blood loss in these situations, it is possible that if it is given to a woman with PPH, it may help to reduce the bleeding. But at the moment we do not know if it will help for PPH or not.

What does the study involve?

If a woman develops postpartum haemorrhage, the doctor will examine her, look at her medical records and decide whether she is suitable for the study. If she is suitable and well enough, the doctor will discuss the study with her and ask if she would be willing to take part in the WOMAN Trial. Otherwise her suitability for the trial will be discussed with her representative or the doctor/midwife primarily responsible for her, to see if she can join the trial.

If she does take part, she will receive an injection of either the TXA or a placebo (a liquid which does not contain TXA) directly into the vein. If after a while the bleeding still does not stop, the doctor may decide to give another injection of the TXA or placebo.

After six weeks, or when the woman leaves hospital, the doctor or midwife will collect some more information from the medical records of the woman and her baby/ies to let the trial team know how she is getting on.

Making a decision

Please discuss this with family and friends and if you need more information the research team at this hospital will be happy to discuss the WOMAN trial with you.

woman
World Maternal Antifibrinolytic Trial

PLEASE CONTACT:

Name of doctor or midwife	
Address	
Telephone	
Email	

Women do not have to make a decision now about taking part in this study. This information sheet is to allow them to consider carefully their wishes in the event they are asked to take part. However, if after reading this and discussing it with others you feel that you definitely do NOT want to be involved in this study, please tell your doctor or midwife and ask them to make a note in your medical records.

The study is organised by the London School of Hygiene & Tropical Medicine (University of London) and you can also contact them directly for information about the trial.

woman

World Maternal Antifibrinolytic Trial

Website	www.womantrial.Lshtm.ac.uk
Address	Trials Coordinating Centre, Room 180<br>London School of Hygiene &<br>Tropical Medicine<br>Keppel Street, London WC1E 7HT<br>United Kingdom
Tel	+44(0)20 7299 4684
Fax	+44(0)20 7299 4663
Email	thewomantrial@Lshtm.ac.uk

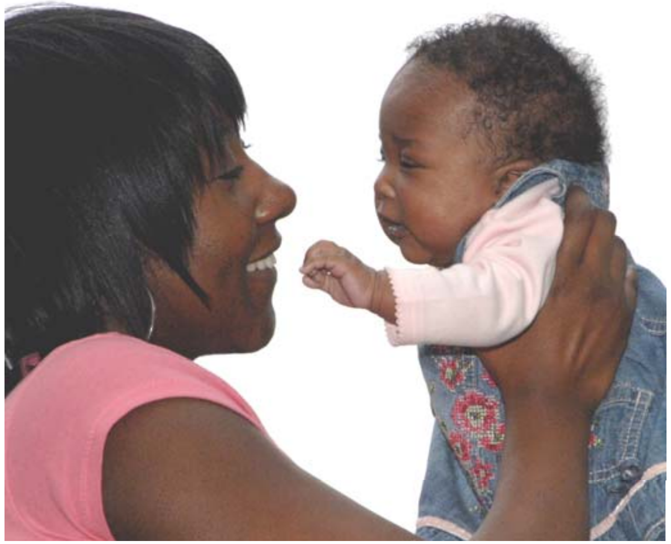

GENERAL INFORMATION LEAFLET ABOUT THE WOMAN TRIAL

woman

World Maternal Antifibrinolytic Trial

Please take a copy
