# Supplementary material for: The WOMAN Trial (World Maternal Antifibrinolytic Trial): tranexamic acid for the treatment of postpartum haemorrhage: an international randomised, double blind placebo controlled trial
Source: Trials. 2010 Apr 16;11:40. doi: 10.1186/1745-6215-11-40 (PMC2864262; doi:10.1186/1745-6215-11-40)
Supplement: Additional file 4 — Form 4. Information sheet for the woman and her representative, pages 1-3. [file 1745-6215-11-40-S4.PDF]

Principal Investigator name, Hospital name
Hospital address
Telephone contact number, email for PI

INFORMATION SHEET FOR THE PATIENT AND HER REPRESENTATIVE(S)

THE WOMAN TRIAL

TITLE OF RESEARCH: TRANEXAMIC ACID FOR THE TREATMENT OF POSTPARTUM HAEMORRHAGE: AN INTERNATIONAL RANDOMISED, DOUBLE BLIND, PLACEBO CONTROLLED TRIAL
TRIAL SITE NUMBER: [ID from database]
LEAFLET VERSION: VERSION 1.0 DATED: 11 MAY 2009

This hospital is taking part in an international research study to find ways to improve the treatment of women who have severe bleeding after delivery of their baby.

(1) We would like to invite you to take part in this study
(2) When you were very unwell you were included in this study and we would like you to continue to take part
(3) As a representative of the patient we are asking you to make a decision on her behalf
(Please circle the option that applies)

The Research Doctor has already checked to make sure you/the patient is medically suitable for this research and you are being asked to make a decision about whether you/the patient can be included in this study. This sheet gives information about the study, including the reasons why the study is being done, and the risks and benefits of taking part.

PLEASE READ THE INFORMATION BELOW CAREFULLY AND ASK THE DOCTOR OR MIDWIFE LOOKING AFTER YOU ANY QUESTIONS YOU MAY HAVE.

1) What is the purpose of the study?

In this hospital, women who have a very severe bleeding after childbirth (also called **postpartum haemorrhage**) are given the best available treatments. The aim of this research study is to see if there is a better treatment for women who have severe bleeding after childbirth. We hope that the treatment (**tranexamic acid**) will help the blood to clot sooner, and so lessen the amount of blood lost and reduce the need for a blood transfusion and other treatments. But it is also possible that the study treatment may cause clots where they are not needed, and because the drug is not routinely used after childbirth, we do not know all the likely side effects. We hope to find that the treatment will do a little more good than harm but we don’t yet know this.

2) Why is this research being done?

Postpartum haemorrhage can be a very serious condition and sometimes requires surgery to control the bleeding. Many thousands of women worldwide die each year from this condition and it is important to find better ways of controlling excessive bleeding after childbirth.

Tranexamic acid is often used to reduce bleeding after major operations such as heart operations. Some women who have heavy menstrual bleeding (periods) also use tranexamic acid. The WOMAN study is being done to see if TXA can reduce bleeding in women with postpartum bleeding.

3) Why have you been invited?

You have been diagnosed with postpartum haemorrhage by your doctor. Your doctor has checked that you are suitable for the study, but it is up to you whether or not you decide to take part.

4) Who is doing the study and who can you call if you have any questions or problems?

Dr ______________________________ is in charge of this study at this hospital. The study is coordinated by doctors and a trial team at The London School of Hygiene & Tropical Medicine (University of London). If you have any questions you can contact the doctor at:

Address:	
Telephone:	

You are also free to visit the trial website to keep up to date with the progress of the trial: www.thewomantrial.Lshtm.ac.uk

5) A patient cannot be in this study if:

- The doctor thinks there is a particular reason why tranexamic acid definitely **should not** be given
- The doctor thinks there is a particular reason why tranexamic acid definitely **should** be given
- They are not an adult

6) What will happen/has happened during this study?

You will be given all the usual emergency treatments for severe bleeding after childbirth, including fluids to replace the blood that you have lost. You will also be given a dose of either the tranexamic acid or a placebo (a liquid which doesn't contain tranexamic acid). This dose will be given as an injection into your vein. If after about 30 minutes you are still bleeding, or if the bleeding stops and starts again within 24 hours after the first dose, you may be given a second dose of the same. You will not receive more than two injections for the study.

We do not know whether giving tranexamic acid on top of all the other treatments will help or not, so half the women in the study will receive tranexamic acid and the other half will receive a placebo. The choice of which treatment you receive is completely random and you will have an equal chance of receiving either one. Neither you nor the doctor treating you will know which treatment you receive. This information is kept on a confidential list at an independent location in London. The study involves no extra tests but your doctor/midwife will send brief details about your treatment and recovery to the Coordinating Centre in London. They will also send information about the health of your baby/ies. If after discharge from hospital and up to 42 days after treatment you develop any medical problems, please let the doctor named on this form know. This information will be used in strict confidence by the people working on the study and will not be released under any circumstances.

7) What are the possible risks of being in the study?

Tranexamic acid is NOT a new drug and it is widely used to reduce bleeding in conditions such as major heart surgery. There is no conclusive evidence of serious side effects with short term use. But the study treatment may cause clots where they are not needed and, because the drug is not routinely used after childbirth, we do not know all the likely side effects. Your doctor will report to the trial organisers any unexpected problems you may have.

8) What are the possible benefits of being in the study?

We hope that tranexamic acid may help reduce blood loss. The knowledge that we gain from this study will help women with postpartum haemorrhage worldwide in the future.

9) What information do we keep private?

All information about you and the reason for bleeding after childbirth will be kept private. The only people allowed to look at the information will be the doctors who are running the study, the staff at the Coordinating Centre and the regulatory authorities who check that the study is being carried out correctly. The Trial Coordinating Centre may want to collect or copy some trial documents which will have your name and will include the signed Consent Form. This will help them to ensure that the trial is being carried out correctly. Your details will remain confidential and will be held in secure storage at the Trial Coordinating Centre. Your confidential information will be kept separately from the trial data and will be destroyed within five years of the trial ending. We will publish the results of the study in a medical journal so that other doctors can benefit from the knowledge, but your personal information will NOT be included and there will be no way that you can be identified.

10) Can you change your mind about being in the study?

You can always withdraw from the study at any time. You just need to say for example *"I've decided I don't want to be in this study now"*. We hope that you will let us use information about how you got on, but if you do not want us to use it please tell the doctor.

11) What else do you need to know?

- In the event that something does go wrong and you are harmed during the study, the London School of Hygiene & Tropical Medicine who are organising the study would be responsible for claims for any non-negligent harm suffered as a result of participating in this study.
- We will ask you to sign a separate consent form and give you a copy to keep and you can also keep this information sheet.
- This study has been reviewed and approved by a Research Ethics Committee.

12) What happens afterwards?

If after you leave this hospital you develop any problems at any time up to 42 days after you had your baby, we would definitely want to know about it. You will be given a card with the contact details of the research doctor at this hospital, which you should keep safely and show to anyone who may be treating you for any illness.

If you would like to have a copy of the final results of this study, please let the research doctor know and s/he will ensure you receive a copy when it is published.
