# Supplementary material for: The WOMAN Trial (World Maternal Antifibrinolytic Trial): tranexamic acid for the treatment of postpartum haemorrhage: an international randomised, double blind placebo controlled trial
Source: Trials. 2010 Apr 16;11:40. doi: 10.1186/1745-6215-11-40 (PMC2864262; doi:10.1186/1745-6215-11-40)
Supplement: Additional file 5 — Form 5. Patient consent form. [file 1745-6215-11-40-S5.PDF]

Principal Investigator name, Hospital name
Hospital address
Telephone contact number, email for PI

CONSENT FORM FOR THE PATIENT

THE WOMAN TRIAL

Title of Research: Tranexamic acid for the treatment of postpartum haemorrhage: An international randomised, double blind, placebo controlled trial

Hospital Code Number		Name of Local Principal Investigator	
Patient Hospital ID Number		Randomisation Number	BOX: ___ ___ ___ ___ PACK: ___ ___
Name of Patient			

Version Number: 1.0 / Version Date: 11 May 2009 **PLEASE INITIAL BOXES**

1. I confirm that I have read and understood the information sheet Version Number_____, version date ____________, for the above study and have had the opportunity to ask questions. ☐
2. I understand that my participation is voluntary and that I am free to withdraw at any time, without giving any reason and without my medical care or legal rights being affected. ☐
3. I understand that sections of my medical notes and those of my baby/ies may be looked at by responsible individuals involved in the study. I give permission for these individuals to have access to these records. ☐
4. I give permission for a copy of this consent form, which contains my personal information, to be made available to the Trial Coordinating Centre in London for monitoring purposes only. ☐
5. I give permission for my personal doctor to be given information about my participation in this trial. ☐
6. I agree to take part in the above study, the WOMAN trial. ☐

______________________	____________	______________________
Name of Patient	Date	Signature / Thumbprint<br>or other mark (if unable to sign)
______________________	____________	______________________
Name of person taking consent	Date	Signature
______________________	____________	______________________
Name of local principal investigator	Date	Signature

(Witness only if required) The patient is unable to sign and as a witness I confirm that the patient has been given all the information about the trial and has verbally consented to taking part.

______________________	____________	______________________
Name of witness	Date	Signature

Original to be filed in the Investigator's Study File, 1 copy for patient,

1 copy to be kept with woman's hospital records
