# Supplementary material for: The WOMAN Trial (World Maternal Antifibrinolytic Trial): tranexamic acid for the treatment of postpartum haemorrhage: an international randomised, double blind placebo controlled trial
Source: Trials. 2010 Apr 16;11:40. doi: 10.1186/1745-6215-11-40 (PMC2864262; doi:10.1186/1745-6215-11-40)
Supplement: Additional file 6 — Form 6. Representative consent form. [file 1745-6215-11-40-S6.PDF]

Principal Investigator name, Hospital name
Hospital address
Telephone contact number, email for PI

CONSENT FORM FOR THE PATIENT'S REPRESENTATIVE

THE WOMAN TRIAL

Title of Research: Tranexamic acid for the treatment of postpartum haemorrhage:
An international randomised, double blind, placebo controlled trial

Hospital code number		Name of Local Principal Investigator	
Patient Hospital ID Number		Randomisation Number	BOX / PACK
Name of Patient			
Name of Representative		Relationship of representative to patient	

Version Number: 1.0 / Version Date: 11 May 2009 **PLEASE INITIAL BOXES**

1. I confirm that I have read and understood the information sheet Version Number________, version date______________, for the above study and have had the opportunity to ask questions.
2. I confirm that I am not aware of any reason why this patient would have objected to taking part in this study.
3. I understand that my consent is voluntary and that I am free to withdraw it at any time without giving any reason and without the patient's medical care or legal rights being affected.
4. I understand that sections of the patient's medical notes and those of her baby/ies may be looked at by responsible individuals involved in the study.
5. I give permission for a copy of this consent form which contains my personal information to be made available to the Trial Coordinating Centre in London for monitoring purposes only.
6. I give permission for the patient's personal doctor to be given information about her participation in this trial.
7. I agree for the above named patient to take part in the WOMAN trial.

____ ______________________

Signature / thumbprint or other mark of Representative (if unable to sign) Date

____________________________ ______________ ____________________________

Name of person taking consent Date Signature

____________________________ ______________ ____________________________

Name of local principal investigator Date Signature

(Witness only if required) The representative is unable to sign and as a witness I confirm that the representative has been given all the information about the trial and has verbally consented to taking part.

____________________________ ______________ ____________________________

Name of witness Date Signature

Original to be filed in the Investigator's Study File, 1 copy for representative, 1 copy to be kept with woman's hospital records
